# Notes

**Published:** 1898-03-12

**Authors:** 


### NOTES.

A sum of £1,000 has been bequeathed by Mr. H. W. Freeman, of Bath, towards the endowment of an obstetric scholarship at the Middlesex Hospital.

An active propaganda is on foot in Rangoon for providing a Chinese hospital, since the poor Chinese there suffer seriously from want of proper medical and nursing attention.

An admirable standard of hospital work is already established in South Africa. The new Somerset Hospital in Cape Town uses the Röntgen rays in diagnosis, and possesses a disinfecting oven which many a London hospital might well envy.

The sum of £360 resulted from a minstrel performance on board the U.S. cruiser *San Francisco* at Nice, and this, with £40 from a lady resident, completes the amount necessary for the endowment of a Jubilee bed in the Victoria Ward of the Foreign Hospital at Nice.

Three new pavilions have been added to the infirmary of the Toxteth district of Liverpool, these bringing up the accommodation at the disposal of the Guardians to 412 beds. The wards of these pavilions are fitted with enamelled brick dados, and muraneze glass in the lower panes of the windows, and are heated by hot-pipes and open stoves.

It has given us pleasure to receive a letter of thanks from the committee of the Royal Free Hospital through the secretary, Mr. Conrad Thies, for the report of the proceedings at their annual meeting which appeared in our columns. We make a point of expressing our gratification on receiving this courteous communication, as we believe that those who administer hospitals are frequently under the impression that sending a representative to report on a meeting is a mere matter of routine in the conduct of a journal, and that no particular interest is evinced by such an act. At any rate, this is not the case in regard to The Hospital. A genuine desire to record the progress and report the necessities of the hospitals induces the editors of The Hospital to open their columns to specially reported notices of hospital meetings that interest in them may be stimulated, and courteous recognition of this effort to assist, such as has reached us from the Royal Free Hospital, is very welcome.

